# Microbiome variations among age classes and diets of captive Asian elephants (*Elephas maximus*) in Thailand using full-length 16S rRNA nanopore sequencing

**DOI:** 10.1038/s41598-023-44981-z

**Published:** 2023-10-17

**Authors:** Worata Klinsawat, Pichahpuk Uthaipaisanwong, Piroon Jenjaroenpun, Supaphen Sripiboon, Thidathip Wongsurawat, Kanthida Kusonmano

**Affiliations:** 1 Conservation Ecology Program, School of Bioresources and Technology, King Mongkut’s University of Technology Thonburi, Bangkok, Thailand; 2 Systems Biology and Bioinformatics Research Group, Pilot Plant Development and Training Institute, King Mongkut’s University of Technology Thonburi, Bangkok, Thailand; 3 Division of Medical Bioinformatics, Faculty of Medicine Siriraj Hospital, Mahidol University, Bangkok, Thailand; 4 Department of Large Animal and Wildlife Clinical Science, Faculty of Veterinary Medicine, Kasetsart University, Kamphaeng Saen Campus, Nakhon Pathom, Thailand; 5 Bioinformatics and Systems Biology Program, Schools of Bioresources and Technology, King Mongkut’s University of Technology Thonburi, Bangkok, Thailand

**Keywords:** Microbiome, Microbial communities, Sequencing

## Abstract

Asian elephant (*Elephas maximus*) is the national symbol of Thailand and linked to Thai history and culture for centuries. The elephant welfare improvement is one of the major components to achieve sustainable captive management. Microbiome inhabiting digestive tracts have been shown with symbiotic relations to host health. This work provided high-resolution microbiome profiles of 32 captive elephants at a species level by utilizing full-length 16S rRNA gene nanopore sequencing. Eleven common uncultured bacterial species were found across elephants fed with solid food including uncultured bacterium *Rikenellaceae RC9 gut group*, *Kiritimatiellae WCHB1-41*, *Phascolarctobacterium*, *Oscillospiraceae NK4A214 group*, *Christensenellaceae R-7 group*, *Oribacterium*, *Oscillospirales UCG-010*, *Lachnospiraceae*, *Bacteroidales F082*, uncultured rumen *Rikenellaceae RC9 gut group*, and *Lachnospiraceae AC2044 group*. We observed microbiome shifts along the age classes of baby (0–2 years), juvenile (2–10 years), and adult (> 10 years). Interestingly, we found distinct microbiome profiles among adult elephants fed with a local palm, *Caryota urens*, as a supplement. Potential beneficial microbes have been revealed according to the age classes and feed diets. The retrieved microbiome data could be provided as good baseline microbial profiles for monitoring elephant health, suggesting further studies towards dietary selection suitable for each age class and the use of local supplementary diets.

## Introduction

Asian elephant (*Elephas maximus*) is the largest land mammal species in the family Elephantidae. Thailand has prioritized elephant conservation and captive management as the animal has been linked to Thai history and culture. As generalist herbivores, elephants can feed on a wide variety of diets including plant leaves, branches, stalks, bark, grass, and roots^1,2^. For husbandry management, the improvement of nutritional provision and enrichment practices is one of the major components to achieve elephant welfare, health, and reproduction^3^. Proper dietary management has always been taken into account as animals in captivity rely on restricted varieties of diets compared to wild animals^4^. A better understanding of the relationships between dietary intake, nutrition, and elephant health will help promote physiological functions and welfare practices.

Microbiome in digestive tracts has been shown with a strong association to host health and intake diets^5–7^. Microbiome inhabiting host’s digestive tracts has played an essential role in aiding digestion^8^, host immune system and overall health^9,10^, neural development and behavior^11^, nutrition absorption^12^, and energy consumption^13^. In herbivores, gut microbiome particularly facilitates plant toxin intake^14^. Microbiome profiles are specific to each individual and could be influenced by different factors, not only diets, but also ages^15,16^, phylogeny^17^, stress level^18^, and geographical locations^19^. Revealing microbiome profiles and associations with different factors is essential for leading an improvement in host health.

Diets have been reported as one of the key factors influencing microbiome variation and health status in elephants and other herbivores. Elephants and other hindgut fermenters have enlarged caecum and colon. Microbial fermentation in this lower digestive tract enables herbivores to gain energy from indigestible plant diets through the degradation of cellulose and hemicellulose into polysaccharides and monosaccharides including glucose and cellobiose^20,21^. The dominant bacteria taxa involved in fermentation processes include Firmicutes (Family Oscillospiraceae), Proteobacteria, and Bacteroidetes, followed by Actinobacteria, Verrucomicrobia, and Fibrobacteres^19^. In Asian elephants, the abundance of Firmicutes and hemicellulose-degrading hydrolases were high in both wild and captive elephants, suggesting a strong digestibility of lignocellulose diet in Asian elephants^22,23^. Moreover, a study in wild African elephants^24^ showed that seasonal variation in food availability is correlated with the change in microbial composition.

The transition from infancy to juvenescence and adulthood is another key factor influencing changes in gut microbiota. Dietary transition from milk to solid food during nursing to weaning periods is associated with the shift in dominant bacteria phyla in captive Asian elephants^13^, domestic pig^25^, and rhesus macaques^26^. The gut microbiome profiles that change along the host’s life stage are considered to be important components of development and homeostasis^27^. Uncovering dietary intake and its effects on host-microbiome relationships across life stages are crucial for monitoring and managing health, fitness, and welfare of captive animals. Beneficial microbes or diet preferences could be chosen for captive elephants at different life stages to benefit the host health conditions^4,28^.

The advent of high-throughput sequencing technologies facilitates the study of the microbial community without culturing^29,30^. Genetic materials of a whole microbial community could be directly sequenced through high-throughput sequencing technologies. Amplicon sequencing of 16S rRNA genes is commonly applied to characterize microorganisms in studied samples^31^. With short-read technologies, one or two-consecutive hypervariable regions are selected for microbiome analysis as the targeted regions are in a length size that the technology allows. These studies provide a reliable resolution to identify different microbes at a taxonomic genus level^32,33^. However, full-length 16S rRNA gene has been shown to provide better resolution to distinguish members in a studied community^34^. Long-read or third sequencing technologies such as Oxford Nanopore sequencing could read long nucleotide sequences covering lengths of 16S rRNA genes. This technology provides a portable option and real-time sequencing to use for a field study^34^ and a species-level identification is possible^35^. The higher resolution of taxonomic identification is often required to obtain explicit information on the beneficial microbes as well as pathogens. The use of nanopore sequencing for full-length 16S rRNA gene-based sequencing is of interest.

Currently, Asian elephant microbiome have been investigated, yet with relatively small numbers of research compared to other hosts. Moreover, the observation in a specific environment and local food that influence microbiome like Thailand was only a preliminary observation^36^. In this study, we aim to provide a species-level identification of microbiome among captive Asian elephants in Thailand by utilizing full-length 16S rRNA gene nanopore sequencing technology. The study included fecal microbiome from baby, juvenile, and adult captive elephants with varying diets collected from three geographic locations across Thailand. The retrieved data are microbiome of captive elephants in a real practice which could reflect baseline microbial profiles in healthy captive elephants. Revealing the microbiota could be invaluable information for elephant welfare assessment leading to a guideline for feed preparation towards the improvement of the captive elephant's health and prevention of diseases.

## Methods

### Elephant fecal samples and DNA collection

**Figure 1:**
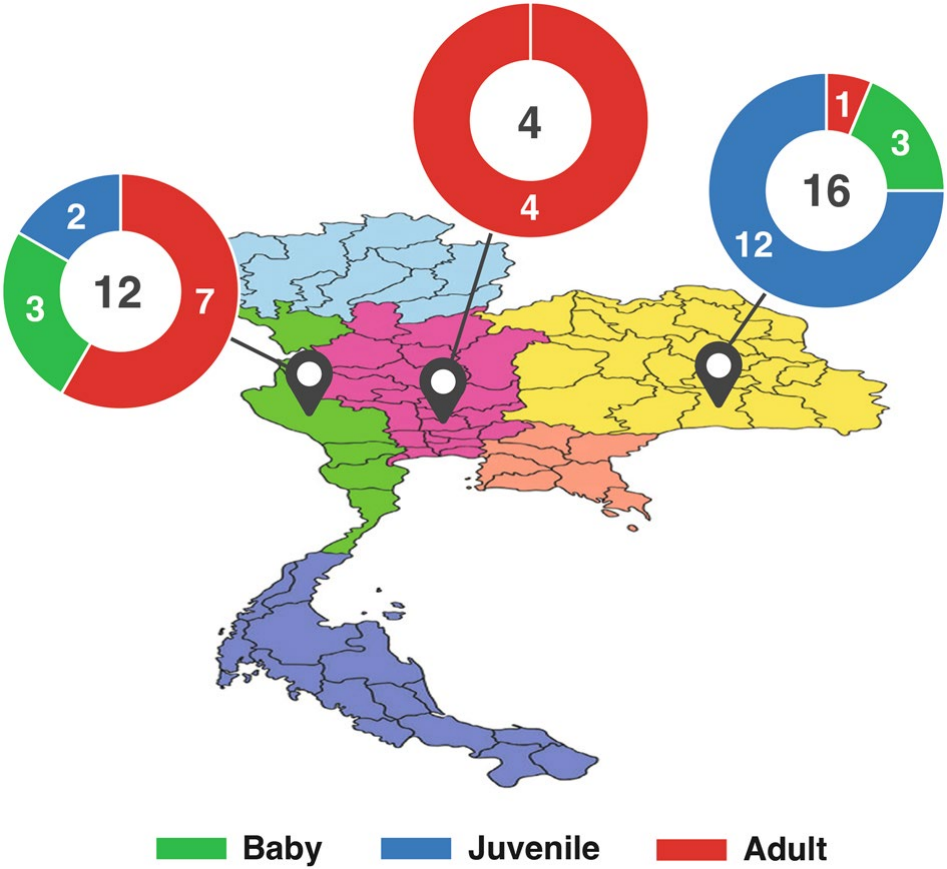
The geographical locations of collected fecal samples of 32 captive Asian elephants in Thailand. Three main elephant camps are located in the west, central and northeast of Thailand. Green, blue and red represent the numbers of fecal samples of baby, juvenile and adult samples, respectively.

A total of 32 fecal samples of captive elephants with varied age classes were collected within 15 min to one hour after defecation from three regions of Thailand: west, central, and northeast (Fig. 1). We classified three age classes: baby (0–2 years), juvenile (2–10 years), and adult (> 10 years)^1^. None of the baby and adult elephants had mother–offspring relationships. Elephant camps were selected due to their husbandry management under the compliance of Thai Elephant Conservation Center and these localities are among the largest, representative of their region. The samples were collected within the dry season to control for bias induced by seasonal diets. Each sample was collected using latex gloves, sterile spoons and 2 mL tubes containing a lysis buffer of ZymoBIOMICS DNA Miniprep Kit (Zymo Research, USA). To prevent cross-contamination, gloves and spoons were discarded after collecting each sample. To overcome impractical transportation method by freezing samples at − 20 °C or − 80 °C and reduce biases in microbial profiling, we collected ~ 200 mg of elephant feces from three random points (both inside and surface) into 750 μl ZymoBIOMICS™ Lysis Solution (catalog number D4300, Zymo Research, USA). Transportation temperature was kept at 4 °C. The lysis buffer has the properties to slow down or prevent microbial growth at either ambient temperature or 4 °C without significant alteration toward bacterial composition^37,38^. Ethics approval and permission for animal sample collection were given by the ethical review board at the King Mongkut’s University of Technology Thonburi (KMUTT-IACUC-2019/022, dated October 3, 2019). The metadata of the elephant fecal samples were shown in Supplementary Table 1.

### DNA extraction and long-read sequencing based on full-length 16S rRNA genes

DNA was extracted from fecal samples using the ZymoBIOMICS DNA Miniprep Kit (Zymo Research, USA), as described previously^36^. DNA concentration and purity were assessed using Nanodrop 2000 (Thermofisher, USA). Extracted DNA was used as the input for targeted-16S rRNA amplification. The 16S Barcoding Kit offers a method of PCR amplifying and barcoding the full-length (~ 1500 bp) 16S rRNA gene from twelve samples and sequencing them together. Libraries for 16S rRNA sequencing were constructed from 20 ng extracted DNA. PCR amplification was conducted using the 16S Barcoding Kit (SQK-RAB204; ONT, UK) and LongAmp™ Taq 2 Master Mix (New England Biolabs, USA). PCR amplification was performed with the following conditions: initial denaturation at 95 °C for 1 min, 25 cycles of 95 °C for 20 s, 55 °C for 30 s, and 65 °C for 2 min, followed by a final extension at 65 °C for 5 min. PCR amplicon (an expected size was 1500 bp) was evaluated with agarose gel electrophoresis, using the Tapestation 2200 (Agilent, USA). PCR products were cleaned up to remove primers, salt, and enzyme using AMPure XP (Beckman Coulter, USA). A total of 100 ng purified amplicons were used for nanopore adapter attachment and sequenced by R9.4/FLO-MIN106 flow cell on a MinION Mk1B sequencer. Each flow cell run was 48-h long.

### Data preprocessing

We converted the Oxford Nanopore Technology (ONT) raw data to sequence data using base-calling and demultiplexing functions of the Guppy v5.0.7 (Oxford Nanopore Technology) with a super accurate model. The base-called reads were preprocessed using Porechop v0.2.4 (https://github.com/rrwick/Porechop) to clean adapter sequences. Reads with a mean quality score of 10 and a minimum read length of 1200 bases were retained using NanoFilt v2.5.0^39^ for the taxonomic classification step. The sequence data were deposited in the GenBank database with BioProject ID: PRJNA982060.

### Taxonomic classification

NanoCLUST^35^ was utilized to perform clustering of similar reads based on uniform manifold approximation and projection (UMAP), and generated the representative consensus sequences of each cluster using Racon^40^ and Medaka^41^. The representative consensus sequences of 32 elephant gut microbiome samples were accounted for calculating abundance and classifying taxonomy based on SILVA138 SSURef NR99 database^42^. The database showed to provide more completed taxonomic ranking information from domain to species levels, and lower misclassification compared to NR-NCBI and 16S-NCBI databases based on the mock community^43^. Note that the representative sequences that do not have species-level annotation were labeled with a higher annotated taxonomic level indicated in parenthesis (G = Genus and F = Family) (Supplementary Table 2). The microbial abundances of 32 elephant gut microbiomes were normalized by scaling with the minimum total reads among all samples as previously performed^44^.

### Diversity analysis

For alpha-diversity analyses, rarefaction curves and diversity indices of observed species richness (Sobs), Chao1, and Shannon were measured using Mothur v1.47.0^45^. Kruskal–Wallis was used to determine statistically significant differences (p-value < = 0.05) between diversity indices of microbiome groups. Taxonomy profiles were performed based on the normalized abundances at species level. Principal coordinate analysis (PCoA) was generated based on Bray–Curtis dissimilarities, and Permutational multivariate analysis of variance (PERMANOVA) was utilized to measure the differences between the microbial community profiles. Differential abundance analysis was performed using Linear discriminant analysis effect size (LefSe)^46^ to identify microbial species having differential abundance between age classes with Linear Discriminant Analysis (LDA) score > = 2 and p-value < = 0.05. Only microbes with relative abundance greater than 3% were considered. The in-house R scripts and ggplot2 package^47^ were used for data visualization. Phylogenetic investigation of communities by reconstruction of unobserved states (PICRUSt2)^48^ was conducted to predict functions of the microbial communities. A nonparametric t-test (p-value < = 0.05) was employed by Statistical analysis of metagenomic profiles (STAMP)^49^ to detect differential metabolic pathways between elephant age classes.

## Results

### The resulting full-length 16S rRNA gene sequences of the studied elephant microbiome

We derived full-length 16S rRNA nanopore sequences of the 32 fecal samples of captive Asian elephants. The average number of raw reads per sample is 320,691 reads while the minimum and the maximum numbers of raw reads are 52,713 and 2,201,843 respectively. After the data preprocessing step, the numbers of average, minimum and maximum are 264,147, 43,419 and 1,850,777 sequences, respectively. The preprocessed sequences have a mean quality score of 13.9 and a minimum read length of 1438 bases, which were utilized for microbiome analysis. The ONT results of 32 fecal samples of captive elephant samples are shown in Supplementary Table 3. Rarefaction curve analysis indicated sufficient sequences to detect microbial taxa in the samples (Supplementary Fig. 1). As the utilized technology provides long-read sequences for 16S rRNA sequencing (containing nine variable regions, V1 to V9), we could identify microbes at the species level.

### Overall microbiome profiles of captive Asian elephants in Thailand

**Figure 2:**
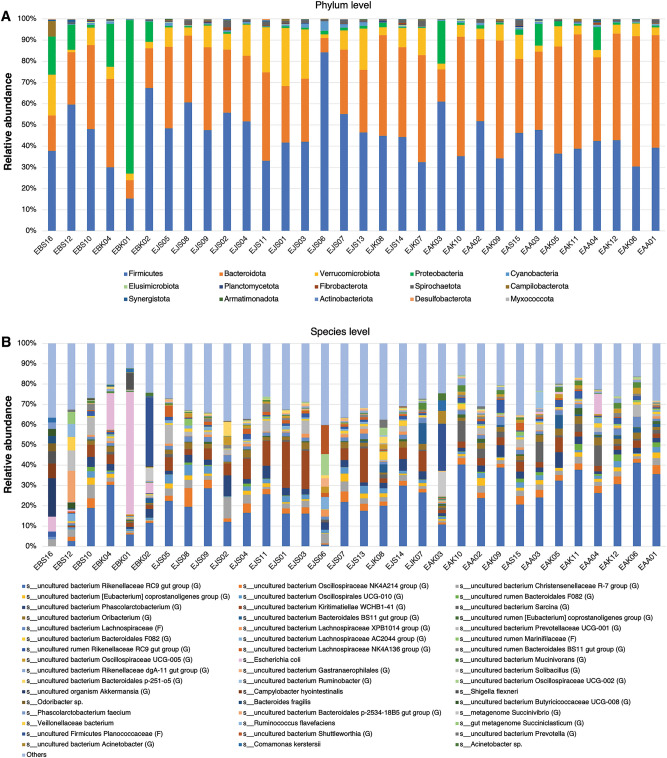
Microbiome profiles at phylum (**A**) and species (**B**) levels of 32 captive elephants in Thailand. The samples were sorted according to the elephants’ ages. At the species level, microbes with abundances greater than 3% are shown.

From the analysis of full-length sequences of 16S rRNA genes, a total of 758 microbial species were observed among 32 fecal samples of captive Asian elephants. The minimum and maximum Sobs are 74 (sample EAK11) and 285 (sample EAA02), respectively. Figure 2A,B show microbiome profiles of 32 captive Asian elephants at phylum and species levels, respectively. Four major phyla of the captive elephant fecal microbiomes are Firmicutes (45.43%), Bacteroidota (36.60%), Verrucomicrobiota (8.71%) and Proteobacteria (6.21%). By sorting the microbiome profiles by age, Firmicutes and Verrucomicrobiota are observed to be more prevalent in juveniles compared to babies and adults. Bacteroidota clearly increased in older elephants. Proteobacteria are more dominant in babies than in juveniles and adults. At species level, eleven bacteria were found in common across 30 elephants fed with solid food (excluding 2 baby elephants fed with only milk) which are uncultured bacterium *Rikenellaceae RC9 gut group* (23.44%), uncultured bacterium *Kiritimatiellae WCHB1-41* (5.98%), uncultured bacterium *Phascolarctobacterium* (3.53%), uncultured bacterium *Oscillospiraceae NK4A214 group* (3.00%), uncultured bacterium *Christensenellaceae R-7 group* (2.91%), uncultured bacterium *Oribacterium* (2.26%), uncultured bacterium *Oscillospirales UCG-010* (2.07%), uncultured bacterium *Lachnospiraceae* (2.00%), uncultured rumen *Bacteroidales F082* (1.05%), uncultured rumen *Rikenellaceae RC9 gut group* (1.45%), and uncultured bacterium *Lachnospiraceae AC2044 group* (0.86%). Note that with the utilized technology, some pathogen species, for example, *Escherichia coli*, *Campylobacter hyointestinalis* (1 out of 6 baby elephants), and *Shigella flexneri* (2 out of 6 baby elephants), were detected. Even though all studied elephants were reported with healthy conditions, the detection would lead to further investigation and awareness of elephant health.

### Different microbiome profiles of captive elephants revealed among age classes and diets

**Figure 3:**
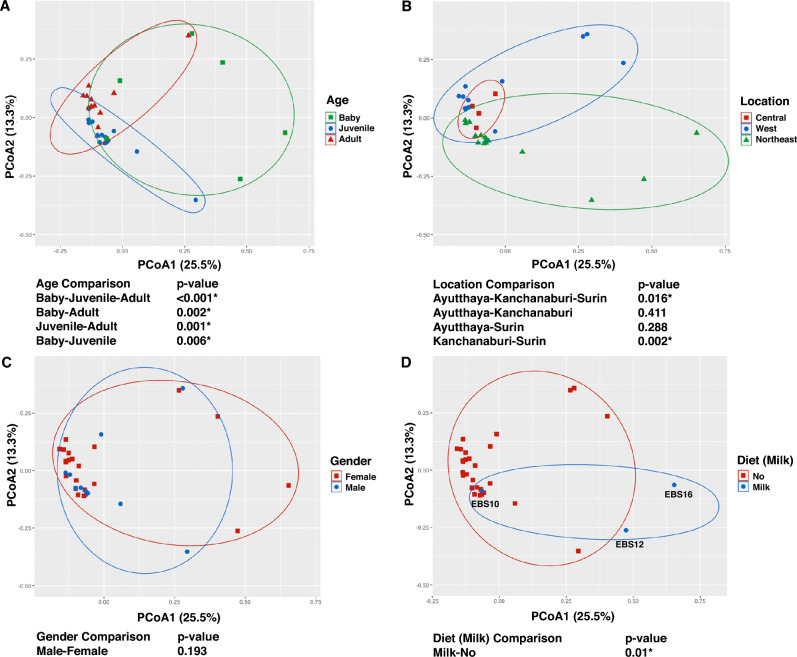
Principle Coordinate Analysis (PCoA) plots based on Bray–Curtis dissimilarity measures of microbial communities of the captive elephant fecal samples. Each PCoA plot is shown with different metadata of the elephants which are age class (**A**), location (**B**), gender (**C**) and diet (**D**), respectively. Permutational multivariate analysis of variance (PERMANOVA) was used to measure the significant differences between different categories of each metadata, which are shown below the plots. The microbial profiles between categories that are significantly different were marked with an asterisk (p-value < = 0.05).

The distances of microbiome profiles based on Bray–Curtis dissimilarity between different age classes, locations, genders and diets were measured. Figure 3 shows PCoA plots of the 32 elephant microbiome samples with different labels of the metadata, which are ages, locations, genders and diets. Statistical significant differences between the microbiome profiles were detected among age classes and diets. By considering both microbial richness and abundance (quantitative measure), significant differences were detected among the age classes of baby vs juvenile vs adult (p-value < 0.001), and between all subgroups which are baby vs juvenile (p-value = 0.006), juvenile vs adult (p-value = 0.001), and baby vs adult (p-value = 0.002) (Fig. 3A). For locations or habitats of the elephants, significant differences could be detected only between west and northeast regions (p-value = 0.002) (Fig. 3B). There is no significant difference between male and female samples (p-value = 0.193) (Fig. 3C).

We provided evidence that diet types influence differences in the microbiome profiles of captive elephants. The difference between microbial profiles of elephants fed with and without milk shows the most significance (p-value = 0.01) (Fig. 3D). Noticeably, two baby elephants (EBS12 and EBS16) fed with milk only show distances of the microbiome profiles apart from a baby fed with milk and banana (EBS10) which have a closer distance to the juveniles. For other diets, we investigated microbiome profile differences among elephants fed with each diet type (i.e. banana, *Caryota urens,* milk, Napier grass, native grass, pineapple, and sugarcane) and without. The microbiome profiles of the captive elephant fed with banana (p-value = 0.02), Napier grass (p-value = 0.008), native grass (p-value = 0.008), and pineapple (p-value = 0.046) were significantly different compared to those fed without (Supplementary Table 4).

### A shift of microbial diversity in the digestive tracts of the captive elephants along the age classes and the corresponding differential abundant microbes

**Figure 4:**
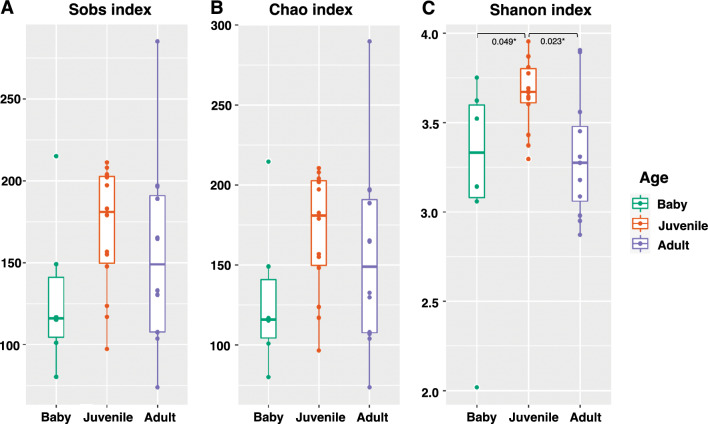
Box plots show alpha microbial diversity indices of baby (0–2 years), juvenile (2–10 years), and adult (> 10 years) elephants, which are observed species richness (Sobs) (**A**), Chao’s diversity index (**B**) and Shannon’s diversity index (**C**), respectively. Statistical significant differences in the diversity indices (Wilcoxon test, p-value < = 0.05) are shown at the top of each box plot.

Microbiome shift of the fecal samples of captive Asian elephants was revealed according to their age classes which are baby (0–2 years), juvenile (2–10 years), and adult (> 10 years). As shown in Fig. 3A, the microbiome profiles of the studied captive elephant are significantly different among the age classes. Figure 4 shows alpha diversity indices between the age classes, which are observed species richness (Sobs), Chao’s diversity index and Shannon’s diversity index. Shannon index, measuring richness and evenness of detected species, shows significant shifts of microbial diversities from babies to juveniles (p-value = 0.049), and juveniles to adults (p-value = 0.023). All indices show the same increasing trend of microbial diversity from babies to juveniles and decreasing trend from juveniles to adults. Microbial profiles showing average relative abundances at family level in each age class are shown in Supplementary Fig. 2.

**Figure 5:**
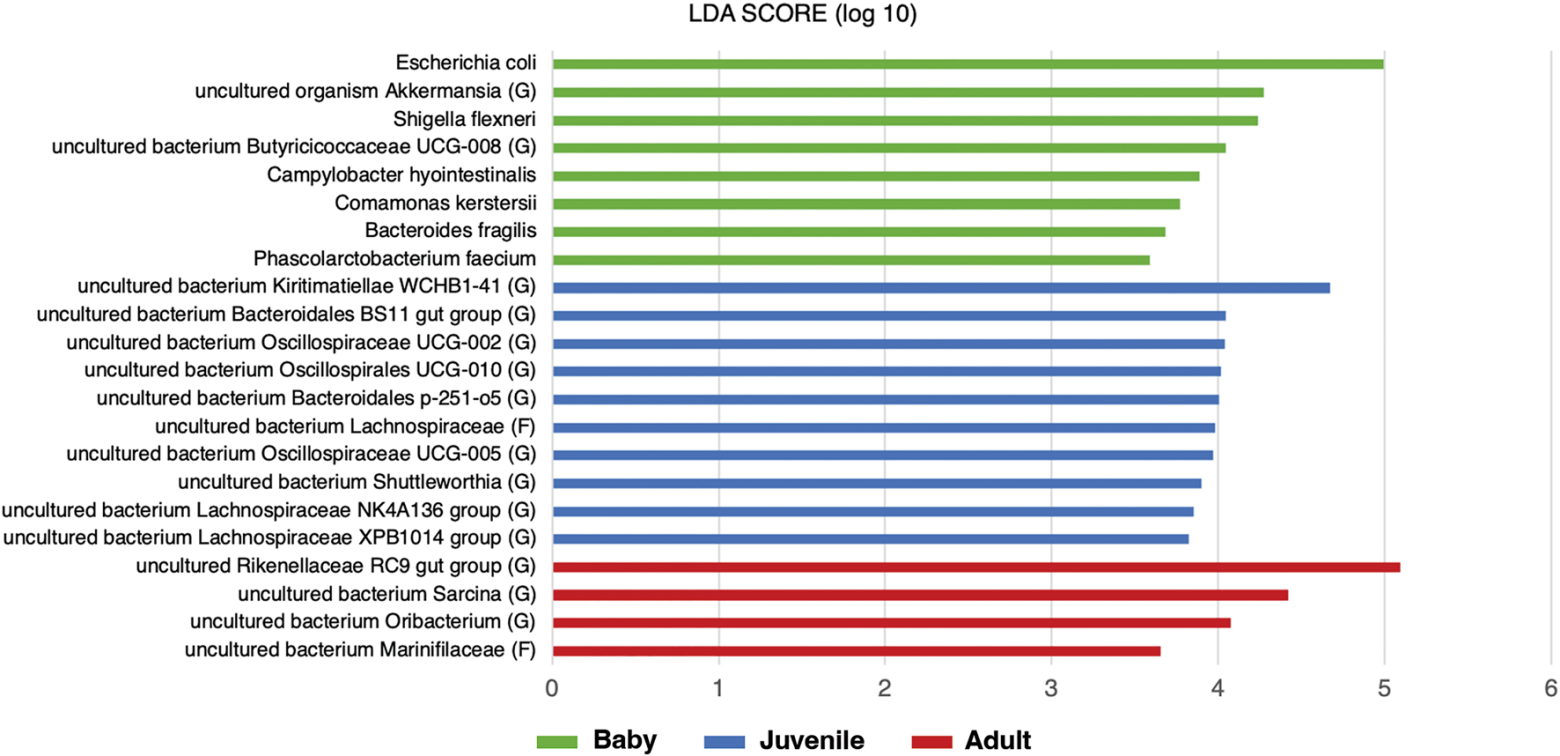
Differential abundant microbes in each age class of captive elephants, which are baby (green), juvenile (blue), and adult (red), respectively.

Differentially abundant microbes between age classes of the captive elephants were identified (Fig. 5). Baby elephants had significantly higher abundances of *Escherichia coli*, uncultured organism *Akkermansia* (G), *Shigella flexneri*, uncultured bacterium *Butyricicoccaceae UCG-008* (G), *Campylobacter hyointestinalis*, *Comamonas kerstersii*, *Bacteroides fragilis*, and *Phascolarctobacterium faecium*, compared to juvenile and adult elephants. *E. coli* had an obviously higher average relative abundance (15.28%) in baby elephants compared to others (0.01% and 1.09% in juveniles and adults, respectively). Juvenile and adult elephants exhibited differential abundances of several uncultured bacteria, including Kiritimatiellae, Bacteroidales, Oscillospiraceae, Lachnospiraceae and Shuttleworthia in juveniles, and Rikenellaceae, *Sarcina*, *Oribacterium*, and Marinifilaceae in adults. For example, uncultured bacterium *Kiritimatiellae WCHB1 41* (G) was dominant in juveniles with a relative abundance of 8.925% (2.53% in babies and 3.33% in adults), whereas uncultured bacterium *Rikenellaceae RC9 gut group* (G) (30.21%) and uncultured bacterium *Sarcina* (G) (4.00%) were prevalent in adult elephants.

### Microbiome profile variations detected in adult captive elephants fed with *Caryota urens*

**Figure 6:**
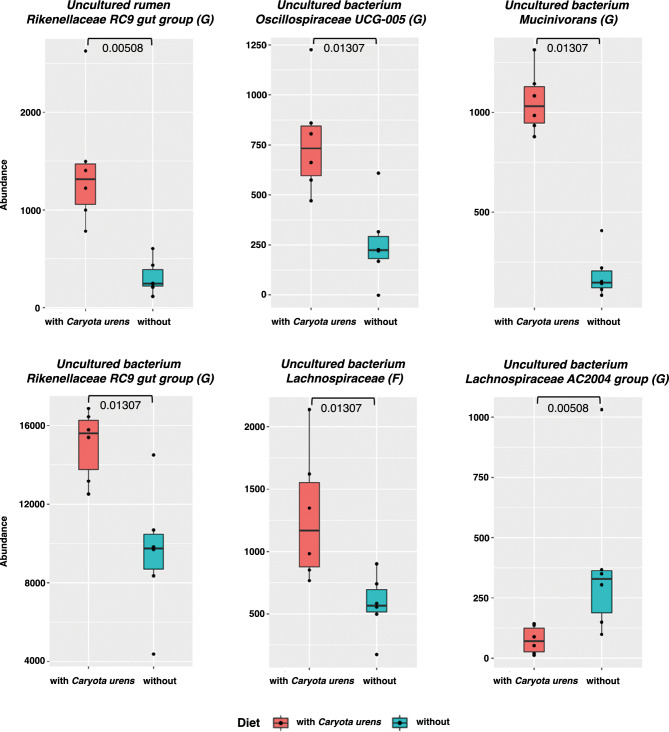
Boxplots display abundances of microbes that are significantly different (p-value < = 0.05) between adult captive elephants in Thailand fed with (n = 6) and without (n = 6) *Caryota urens* as a supplement.

As we observed a shift of the captive elephant microbiome across age classes, we then investigated the microbiome differences of elephants fed with different diets within each age class. Regarding their diets, there was no statistically significant difference in the microbiome profiles of the baby and juvenile groups (Supplementary Table 4). Interestingly, in adult elephants, we found significant differences between the microbial profiles of those fed with *Caryota urens*, a local plant in a palm family, as supplements. We observed that the adult elephants that were not fed with *Caryota urens* were all fed with Napier grass (*Pennisetum purpureum*) as a part of the main diets while those fed with *C. urens* did not consume Napier grass. We detected certain microbes that are significantly different in abundance between these two groups: with vs. without *C. urens* (Fig. 6). Specifically, we identified five microbes that showed significantly higher abundance in elephants fed with *C. urens* as a supplement (uncultured rumen *Rikenellaceae RC9 gut group* (G), uncultured bacterium *Oscillospiraceae UCG-005* (G), and uncultured bacterium *Mucinivorans* (G)*,* uncultured bacterium *Rikenellaceae RC9 gut group* (G), uncultured bacterium Lachnospiraceae (F)), while only one microbe (uncultured bacterium *Lachnospiraceae AC2044 group* (G)) shows higher abundance in the elephants that were not fed with *C. urens*. Moreover, three uncultured bacterium taxa were unique to the Napier-feeding group including *Solibacillus*, Planococcaceae, and *Acinetobacter* (Supplementary Fig. 3).

### Enriched microbial metabolic pathways of the captive elephants in each age class

Differential microbial metabolic pathways (p-value < = 0.05) of the captive elephants were identified for each age class (Supplementary Table 5). Enriched pathways in the baby compared to juvenile elephants are mainly in the metabolism class, and the top three are nitrogen metabolism, phosphonate and phosphinate metabolism, and steroid hormone biosynthesis. Interestingly, the dioxin degradation pathway was also enriched in the baby group. In juvenile elephants, the majority of the enriched pathways appear in both metabolism and genetic information processing classes. The pathways in the genetic information processing class include ribosome, aminoacyl-tRNA biosynthesis, protein export, mismatch repair, homologous recombination, DNA replication, and nucleotide excision repair. It is noteworthy that several differential pathways enriched in juvenile elephants compared with both baby and adult elephants are in biosynthesis metabolism processes, which are valine, leucine and isoleucine biosynthesis, phenylalanine, tyrosine and tryptophan biosynthesis, lysine biosynthesis, peptidoglycan biosynthesis, N-Glycan biosynthesis, pantothenate and CoA biosynthesis, terpenoid backbone biosynthesis, zeatin biosynthesis and streptomycin biosynthesis. Nevertheless, the majority of enriched pathways in adult elephants are associated with degradation metabolism processes such as benzoate degradation, synthesis and degradation of ketone bodies, lysine degradation, valine, leucine and isoleucine degradation, and geraniol degradation.

## Discussion

### Utilization of long-read technology provides high-resolution microbiome profiles at species level

It is now common practice to use 16S rRNA amplicon-based sequencing to identify bacteria in samples being studied. The method has allowed us to identify or capture of non-culturable bacteria across broad taxonomic levels. Our previous data of fecal microbial diversity among baby (n = 4) and adult elephants (n = 4) from different regions of Thailand was performed using short-read sequencing, which was implemented on the Illumina^®^ platform^36^. A hypervariable region of variable region 4 (V4) was selected for microbiome analysis because the targeted regions are within the technology's capabilities. However, the short-read 16S rRNA analysis is largely confined to genus-level resolution at best. In this work, to classify bacteria at species level, nanopore-based full-length 16S rRNA (contains nine variable regions, V1 to V9) information is more clearly distinguished, providing higher resolution identification at the species level.

Although the full-length 16S sequence provides better resolution for bacterial identification, it is important to note that the forward primer (27F) specific for the 16S rRNA gene amplification could not target archaeal 16S rRNA gene^50^. Therefore, in this study, we monitor only the bacterial domain not the archaeal domain. Our previous work showed that the majority of the microbial community (> 97%) in both babies and adults was bacteria, not archaea. The approach of using the commercial full-length 16S Barcoding Kit (SQK-RAB204) available from ONT has a limited ability to detect *Bifidobacterium*^51^. This led us to ask whether *Bifidobacterium* was a dominant or important species in elephants. A recent study reported that *Lactobacillales* but not *Bifidobacteriales* is the dominant lactic acid bacteria found in Asian elephants^52^. Nonetheless, *Lactobacillales* have been found in our samples but not as dominant taxa. Similarly, relative abundances of *Bifidobacterium* in Forest elephants were also low (< 1%) based on the V3-V4 regions of 16S rRNA gene sequences^5,53,54^.

### Microbiome profiles of healthy captive elephants in Thailand were revealed

This study revealed baseline fecal microbiomes of healthy captive Asian elephants in Thailand. The dominance of phyla Firmicutes and Bacteroidota in our study is consistent with previous studies on semi-wild Asian elephants from southwestern China^22^, captive Asian elephants from North American zoos^13,55^, and wild African elephants^19^. The dominant phyla in our study are also consistent with those found in hindgut herbivores including horses^56^ and black rhinoceros^57^. At the family level, the dominance of Lachnospiraceae and Rikenellaceae in adult and juvenile elephants in our study are similar to other studies in captive Asian elephants^13^. Although dominant bacterial genera were found to fluctuate across ages and diet types, those with high relative abundance likely play an important role in metabolic pathways including cellulose degradation and reproductive hormones.

### A better understanding of changes in microbiome profiles across age classes is crucial in providing guidelines for dietary programs to meet nutritional needs and maintain healthy status in captive elephants

In this study, we observed changes in microbiome profiles from baby, juvenile to adult elephants. The varying microbial diversity with age is aligned with microbiome changes during development and homeostasis in humans^58,59^ and other animals^60^. We observed several bacteria establish and became dominant in different age classes, which may benefit the host's health in each life stage. For example, an increase in the relative abundance of the Family Rikenellaceae (Phylum Bacteroidota) and Family Oscillospiraceae (Phylum Firmicutes) (Fig. 2 and Supplementary Fig. 3) from baby to adult elephants was reported to correlate with the dietary shift from milk to solid food in Asian elephants^13,22^ and humans^61^. After the weaning period, the gut microbiota involved in digesting milk oligosaccharides decreases^62,63^ and bacteria that degrade the complex plant polysaccharides and fibers have become more abundant, promoting nutrient-use efficiency from plant-based diets in herbivore^63^. Rikenellaceae and Oscillospiraceae are crucial for plant cell wall degradation through the process of cellulose hydrolysis with Glycoside Hydrolases (GHs) activities^64^. Adult individuals rely on the GHs enzyme family to convert indigestible plant carbohydrates including cellulose, hemicellulose and lignin into short-chain fatty acid (SCFAs) metabolites such as acetate, propionate, and butyrate. SCFAs are important for energy consumption, reducing inflammation and promoting gut barrier integrity^65^. Moreover, the differential metabolic pathway results showed several enriched pathways in adult elephants related to degradation metabolisms which could involve activities to break down complex plant carbohydrates. Notably, *Rikenellaceae RC9 gut group* (Fig. 5) has been associated with health benefits. Previous studies showed a positive correlation between an increase in fiber content in the diet of adult yaks^66^ and dairy cows^67^, and the higher relative abundance of *Rikenellaceae RC9 gut group*. In juveniles, the dominant family Kiritimatiellae^68^ and Lachnospiraceae^69^ documented from our study were also associated with promoted lignocellulose-degrading activities and sugar-utilizing capacity. Specifically, *Kiritimatiellae WCHB1-41*, which was predominant in our juvenile elephants, has also been characterized as the dominant genus in hindgut fermenters such as horses^70^ and lemurs^71^.

### Prevalent microbes in healthy baby elephants might be candidate beneficial microbes and indicators for good health

Dominant microbes in baby captive Asian elephants and evidenced as beneficial microbes in other hosts were revealed in our study (Fig. 5). For example, *Akkermansia* (Phylum Verrucomicrobiota) is a mucin-degrading bacteria residing in a mucus layer of the gastrointestinal tract in humans and a wide variety of animals including horses, pigs, donkeys, rabbits, and rodents^72,73^. *Akkermansia* spp. are present since the first year of human infants^73,74^. *A. muciniphila* has been proposed to be a biomarker for healthy gut microbiome as it has beneficial effects by reducing intestinal inflammation, pathogenic microbial growth, and obesity^75–77^. Information on changes in relative abundance and the role of *Akkermansia* in modulating metabolism and immune response and overall health of elephants and other herbivores is limited. In African elephants, *Akkermansia* spp. could be involved with pathways modulating prolactin concentrations as *Akkermansia* spp. abundance was positively correlated with prolactin^55^, an enzyme that increases during pregnancy and lactation to facilitate milk. The function of *Akkermansia* spp. in gastrointestinal homeostasis and associated health conditions remains unclear and further investigation is needed to elucidate its potential benefits on elephant health.

Another potential probiotic bacteria is the non-enterotoxigenic *Bacteroides fragilis* (NTBF). Although *B. fragilis* was prevalent in our baby elephants, it remains uncertain whether the species belongs to the NTBF or the enterotoxigenic *B. fragilis* (ETBF) subtype, which is pathogenic and linked to diarrheal disease^78^. Conversely, the NTBF subtype has been shown to reduce gastrointestinal inflammation^79,80^ and potentially prevent the development of neurological disorders^80,81^. Further molecular assay is needed to identify whether *B. fragilis* found in our study belongs to the beneficial NTBF and could be one of the candidates of probiotic therapy.

The detection of *Phascolarctobacterium faecium* (phylum Firmicutes) in baby elephants is consistent with the colonization of the gastrointestinal tract in the early life of humans^82^. *P. faecium* is a Gram-positive, anaerobic bacteria and its detection in healthy humans is associated with cellulose digestion and maintenance of metabolic homeostasis^82^ as *Phascolarctobacterium* genus converts succinate to SFCAs including acetate and propionate^83^. Moreover, high relative abundance of *P. faecium* is positively associated with healthy individuals with exercise, physical activities and reduction of obesity in humans^82,84^. Further study is needed to better understand the mechanisms underlying how *P. faecium* has an impact on elephant metabolism.

### Detection of pathogenic species is possibly an early warning of pathogen exposure in captive elephants

In this study, we observed pathogenic species mainly in baby elephants including *Escherichia coli*, *Campylobacter hyointestinalis*, and *Shigella flexneri*. Of the family Enterobacteriaceae, *E. coli* has diverse phylogroups that have the potential to be either commensal or pathogenic^85^. For the mutual relationships, *E. loci* is linked to vitamin K production and resistance pathogen colonization^86^, while virulent strains would cause severe diarrhea. In human infants, *E. coli* is one of the first bacteria colonizing mucus of gut epithelial cells, shredded into intestinal lumen and excreted in feces^87^. For pathogenic strains, wild baby African elephants born around the same time were found to be infected with genetically more similar *E. coli* than those of different ages^88^.

In addition to *E. coli*, our detection of the pathogenic *Shigella flexneri* and *Campylobacter hyointestinalis* suggests early exposure to pathogens and raises concerns for public health. Of the *Shigella *spp., *S. flexneri* is the dominant cause of fever, diarrhea and related mortality in children in developing countries^89^. In mammals, *Shigella* spp*.* and *Campylobacter* spp. were found in waterholes and bone marrow from elephant carcasses in India^90^. In addition to elephant-to-elephant routes, these bacteria in our study could be transmitted from keepers to elephants through direct contact, fecal-contaminated water or soil environments. Transmission of *Campylobacter *spp. among livestock, wild boars, and Iberian ibex through direct contact with the animals, their feces of contaminated environment was also documented^91^. An early detection of zoonotic pathogens in captive animals highlights the potential use of gut microbiome analysis as a noninvasive approach for clinical diagnosis, active disease surveillance, and public health management.

Apart from pathogen detection, the dioxin degradation pathway was found enriched in baby elephants. Dioxin is a toxic chemical that stays in the food chain and environment for a long time and is harmful to humans. The dioxin contaminations were found in the surface soils, rivers, and river sediments^92^. One of the most dioxin release source is the burning of agricultural solid wastes^93^. The west and northeast elephant camps are located nearby agricultural areas and rivers. Accordingly, it has the possibility that elephants received the toxins by eating or drinking contaminated food and water. Finding the dioxin degradation pathway in baby elephants showed the potential route of dioxin transmission from mother to baby elephant via breastfeeding as in human^94,95^. However, there is limited report data on dioxins in Thailand. Thus, dioxin monitoring and awareness of the dioxin contamination transfer to elephants are needed.

### Feeding *Caryota urens* revealed distinct abundances of potential beneficial microbes in adult captive elephants

Wild Asian elephants predominantly feed on grasses, shrubs, herbs, followed by lianas, wild palms, wild bananas, leaves and bark of certain tree species^2^. However, in captivity, they have access to a more limited variety of cultivated crops including grass, hay, and browse items^1,96^. In Thailand, captive elephants’ primary diets vary among camps, but consist of Napier grass, pineapple stalk, corn stalk, bamboo grass, and banana trunk^3,97^. Supplementary foods include banana, sugarcane, pumpkin, watermelon, cucumber, and other local grasses and herbs (Klinsawat, personal observation). In our study, mahouts in western Thailand feed juvenile and adult elephants with leaves and pulped trunks from *Caryota urens*, a flowering plant from the palm family Arecaceae. Local Thai wisdom suggests that *C. urens* might have medicinal properties in alleviating fatigue, inflammation and indigestion. Bloating and chronic constipation are the major health issues in captive populations of adult and older elephants in Thailand (Sripiboon, personal communication). In our study, adult elephants fed with *C. urens* had significant differences in bacterial profiles with higher relative abundances of *Rikenellaceae RC9 gut group* II and III*,* Lachnospiraceae, *Oscillospiraceae UCG-005* (G) II, and mucin-degrading *Mucinivorans.* Studies have shown that the family *Rikenellaceae,* Oscillospiraceae^98^ and *Lachnospiraceae*^69^ are involved in mechanisms that break down the complex plant carbohydrates into the digestible SCFAs. In humans with high-fiber diets, these bacteria families play a crucial role in leanness^14^ by reducing body fat, maintaining metabolic homeostasis, and mediating immune responses. Similar to the perceived benefits by the locals, products of bacterial fermentation in the gut of *C. urens*-feeding group might influence diversity and composition of the healthy gut microbiome and lower the risks of metabolic disorders. Within the family *Oscillospiraceae*, *Oscillospira* is advocated for being a candidate for next-generation probiotics^98^ due to its properties in reducing obesity via production of SCFAs including butyrate. Due to the complex interactions between host and bacteria communities, further study is needed to elucidate underlying mechanisms of interactions among gut bacteria and develop therapeutic strategies for alleviating host metabolic disorders. Overfeeding with *C. urens*, particularly with old trunks containing a lot of gum and mucilage, and less than optimal water intake can cause digestive problems such as bloating and constipation (Sripiboon, personal communication). To specify optimal levels and duration of *C. urens* supplementation, further investigation is needed to determine the safety, efficacy and therapeutic roles of this medicinal plant and other traditional herbal additives on elephant health. In addition, the local palm in Indonesia which is the same Arecaceae family as *C. urens* has been used as a supplement for energy booster in livestock management^99^. Herbal supplement based on local plants was also reported to be given to older elephants in northern Thailand^3^. Moreover, in some camps near natural forested areas, elephants are able to forage the local plants in a semi-captive environment during the night, therefore a variety of diets is likely underreported, leading to bias in the association between diet and microbiome profile variation. A systematic recording of local wisdom and science-based benefits of local herbal plants is needed to improve the nutrition management of captive animals.

Adult elephants fed with Napier grass as a primary diet without *C. urens* herbal supplement also had unique gut microbiome profiles. *Lachnospiraceae AC2044* was enriched, along with three unique taxa of *Solibacillus*, Planococcaceae and Acinetobacter. Due to high yielding, drought tolerance, higher crude protein content compared to other grass varieties, Napier grass and its different cultivars have been used as the primary forage grass for elephants^100^. The enriched butyrate-producing *Lachnospiraceae AC2044* in our Napier-feeding elephants is consistent with improved fiber digestibility in yak^101^ and growth development in horse^102^. However, *Lachnospiraceae AC2044* was also dominant in stressed horses^103^ and reduced cellulose utilization in yaks^104^. In addition to diet and age factors, we need to quantify the feeding level, dietary fiber content, crude protein content, stress hormone, the duration and intensity of exercise to better understand interactions between complex bacterial communities, welfare conditions, and elephant health. Similarly, *Solibacillus* members have shown varied effects on ruminant and human health. *Solibacillus* was found in resource-restricted African buffalos^7^. It is possible that some *Solibacillus* spp*.* are able to adapt during diet variability and subsequently dominate gut microbial community. *Solibacillus* enrichment has been linked to positive health outcomes with no history of gastrointestinal disorders in cattle^105^ and reduced anxiety in mice^106^. An increased stress hormone and diet shift in previous Asian elephant study might be associated with changes in relative abundance of *Planococcaceae*^107^, which is commonly found in our Napier-fed adult elephants, healthy juvenile and adult Asian elephants^107^, and African megafauna^24^.

In adult and aging elephant populations, constipation, inflammation, and increased susceptibility to infection are the major health challenges. A better understanding of the relationships between diet, health condition and changes in gut microbiome composition has become a priority research for elephant nutrition and welfare. Monitoring changes in microbial profiles throughout life history will provide insight into potential medical intervention and nutrition management suitable for each age class. It is noted that this study is of an observational nature, as we examined differential microbiome diversity among selected cohorts without incorporating a control group. Elephants naturally fed on available plants at each camp, therefore restricting our ability to control diet variables or other confounding factors that are intrinsic to observational studies and might affect microbiome profiles.

While this study provides valuable insights into the microbiome profiles of captive elephants in different age groups, it is important to acknowledge the limitation imposed by the relatively small sample size of baby fecal microbiome samples. Further, a larger sample size with a control group will increase the statistical power of the association and might reveal more variations within and between groups, offering a more comprehensive understanding of the microbiome dynamics in these animals. Future studies with expanded sample sizes are necessary to validate and extend the findings presented here, potentially unveiling more complex interactions and associations within the elephant microbiome.

## Conclusion

This study provided a high-resolution analysis of the captive elephant microbiome in Thailand at a taxonomic species level by utilizing full-length 16S rRNA gene nanopore sequencing technology. We observed a total of 32 fecal microbiome samples and found microbiome shifts mainly among age classes and feed diets. Based on our observations, different microbes became dominant in different age classes of baby (0–2 years), juvenile (2–10 years), and adult (> 10 years). As also revealed in other herbivores, the dietary shift from milk to solid food could have microbes involved in digesting milk oligosaccharides decrease, and microbes that degrade the complex plant polysaccharides and fibers have become more abundant, promoting nutrient-use efficiency from plant-based diets. Shifting from baby to juvenile showed a period of microbiome establishment characterized by a dietary shift from milk to plant-based foods. In addition, pathogen species were also observed frequently in baby elephants. Even though all studied elephants were reported as being in healthy conditions, the detection prompts the need for further investigation and increased awareness of elephant health. Enhanced hygiene and welfare management could be a necessary protocol in preventing pathogen infection in baby elephants. Diets promoting beneficial microbes utilizing plant carbohydrates into SCFAs in juveniles and adult elephants could be essential. Several enriched pathways in adult elephants related to degradation metabolisms could involve in breaking down complex plant carbohydrates. Interestingly, we found several taxa dominant in adult elephants fed with local plants, *Caryota urens,* as a supplement. The microbes have been previously reported with symbiotic relations to host health, converting dietary fibers into SCFAs. Our findings support the potential advantages of increased exposure to local plants as supplementary diets and highlight the benefit of incorporating a variety of dietary choices into common husbandry practices. This study provides the very first microbiome of captive elephants at species level, establishing a robust baseline for microbial profiles that can aid in monitoring elephant health. Furthermore, it suggests the consideration of the use of local supplementary diets, and provides microbiome-based evidence for the established feeding practices and welfare management. Further experimental studies into the interactions among gut microbiota and how they are influenced by diet, environment, or other factors will provide insight into the complex processes regulating energy, metabolic homeostasis, and inflammatory responses. A better understanding on these underlying mechanisms could overall health benefits by monitoring related microbes and selecting feed benefiting the gut microbiome.

### Supplementary Information

Supplementary Information.

## Data Availability

The microbiome data of this study is available at the GenBank database with BioProject ID: PRJNA982060, https://www.ncbi.nlm.nih.gov/bioproject/PRJNA982060.
