# Supplementary material for: Microbiome variations among age classes and diets of captive Asian elephants (Elephas maximus) in Thailand using full-length 16S rRNA nanopore sequencing
Source: Sci Rep. 2023 Oct 17;13:17685. doi: 10.1038/s41598-023-44981-z (PMC10582034; doi:10.1038/s41598-023-44981-z)
Supplement: Supplementary file 1 — Supplementary Information. [file 41598_2023_44981_MOESM1_ESM.pdf]

Supplementary Figures

Supplementary Figure 1. Rarefaction curves of the 32 fecal microbiome samples of captive elephants in Thailand. The x-axis represents the number of sequences, and the y-axis represents a measure of the number of observed species. Green, blue and red represent the numbers of baby, juvenile and adult samples, respectively

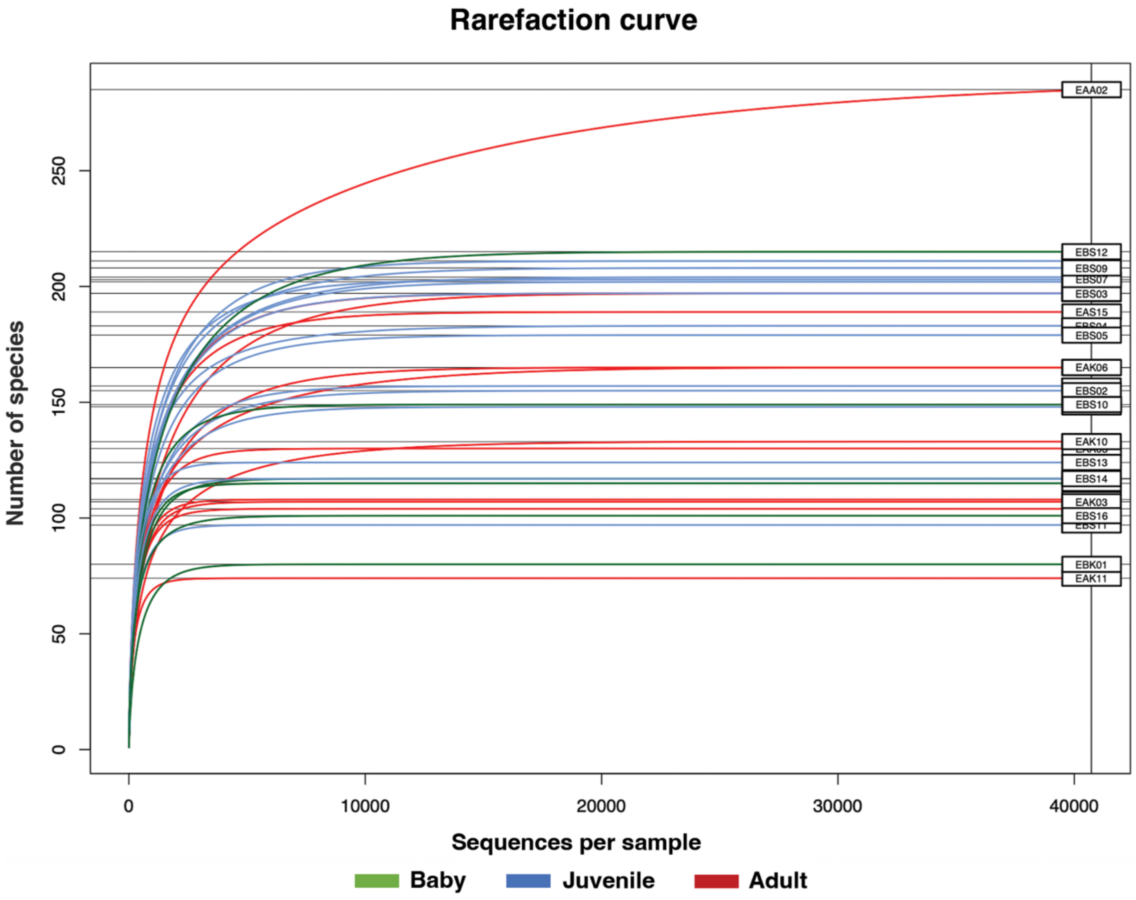

Supplementary Figure 2. Microbial profiles showing average relative abundances (Family level) of fecal microbes in captive elephants from Thailand for each age class of baby (0-2 years), juvenile (2-10 years), and adult (>10 years).

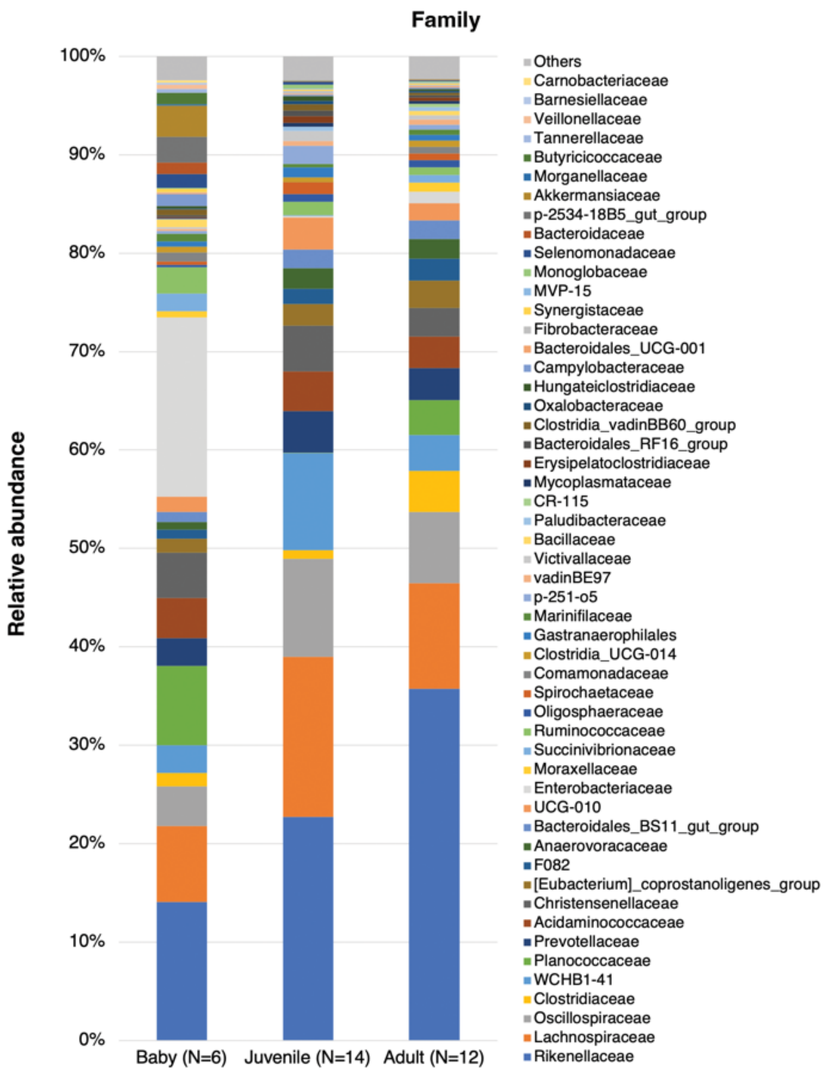

Supplementary Figure 3. Microbial profiles showing average relative abundances (Family level) of fecal microbes in adult captive elephants from Thailand fed with and without Napier grass as a part of the main diet.

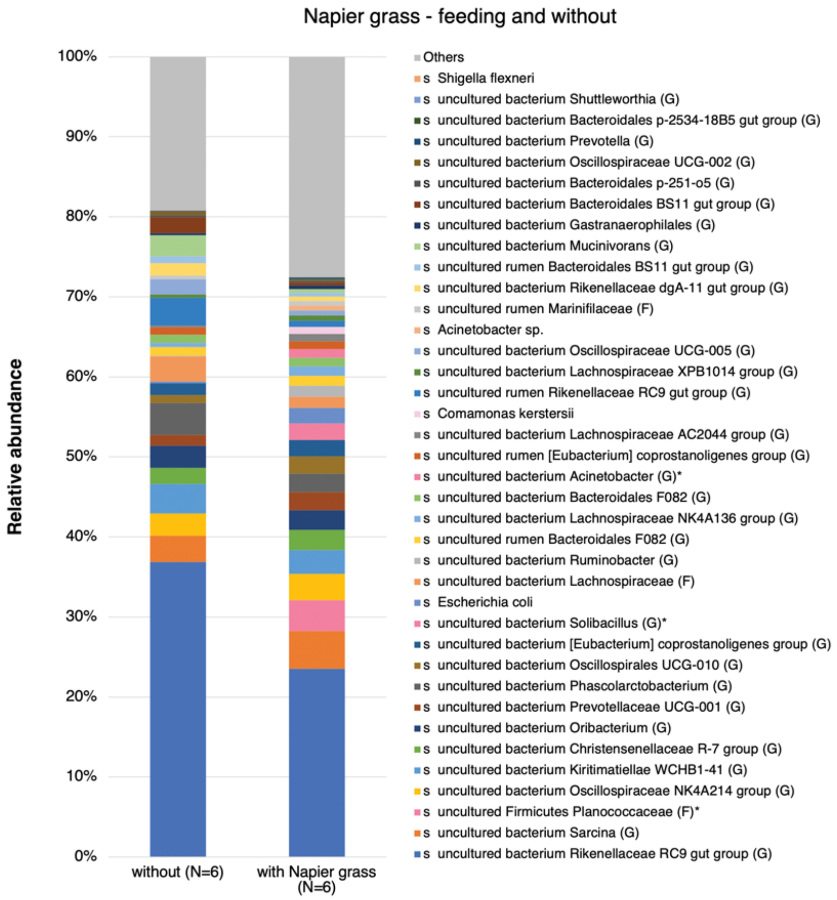

Supplementary Tables

Supplementary Table 1. The metadata of fecal samples of the 32 captive Asian elephants in Thailand. Age classes were classified to baby (0-2 years), juvenile (2-10 years), and adult (>10 years).

Sample	Location	Sex	Age	Napier grass	Banana	Native grass	Pineapple	*Caryota urens*	Sugarcane	Milk
EAA01	Central	Female	Adult	yes	yes	yes	no	no	no	no
EAA02	Central	Female	Adult	yes	yes	yes	no	no	no	no
EAA03	Central	Female	Adult	yes	yes	yes	no	no	no	no
EAA04	Central	Female	Adult	yes	yes	yes	yes	no	no	no
EAK03	West	Female	Adult	yes	yes	no	yes	no	no	no
EAK05	West	Female	Adult	no	yes	no	yes	yes	no	no
EAK06	West	Female	Adult	no	yes	no	yes	yes	no	no
EAK09	West	Female	Adult	no	yes	no	yes	yes	no	no
EAK10	West	Female	Adult	no	yes	no	yes	yes	no	no
EAK11	West	Female	Adult	no	yes	no	yes	yes	no	no
EAK12	West	Female	Adult	no	yes	no	yes	yes	no	no
EAS15	Northeast	Female	Adult	yes	no	no	yes	no	no	no
EBK01	West	Female	Baby	no	yes	no	no	no	yes	no
EBK02	West	Male	Baby	yes	no	no	no	no	yes	no
EBK04	West	Male	Baby	no	yes	yes	yes	no	no	no
EBS10	Northeast	Male	Baby	no	yes	no	no	no	no	yes
EBS12	Northeast	Female	Baby	no	no	no	no	no	no	yes
EBS16	Northeast	Female	Baby	no	no	no	no	no	no	yes
EJK07	West	Female	Juvenile	no	yes	no	yes	yes	no	no
EJK08	West	Female	Juvenile	no	yes	no	yes	yes	no	no
EJS01	Northeast	Male	Juvenile	yes	no	no	yes	no	yes	no
EJS02	Northeast	Male	Juvenile	yes	no	no	yes	no	no	no
EJS03	Northeast	Male	Juvenile	yes	no	no	yes	no	no	no
EJS04	Northeast	Female	Juvenile	yes	no	no	yes	no	no	no
EJS05	Northeast	Male	Juvenile	yes	yes	no	yes	no	yes	no
EJS06	Northeast	Male	Juvenile	yes	yes	no	no	no	no	no
EJS07	Northeast	Female	Juvenile	yes	no	no	yes	no	no	no
EJS08	Northeast	Female	Juvenile	yes	no	no	yes	no	no	no
EJS09	Northeast	Female	Juvenile	yes	yes	no	no	no	no	no
EJS11	Northeast	Male	Juvenile	yes	yes	no	no	no	yes	no
EJS13	Northeast	Male	Juvenile	yes	yes	no	yes	no	no	no
EJS14	Northeast	Male	Juvenile	yes	yes	no	no	no	no	no

Note: Pineapple includes fruits, stems, and leaves.

Supplementary Table 2. Examples of species annotation provided in this study that are labeled with higher taxa levels. The annotated taxonomy from SILVA138 SSURef NR99 database was available with taxonomic annotation from domain to species levels. However, the ambiguous annotations were labeled with higher annotated taxonomic names indicated in parenthesis (G = Genus and F = Family) to provide more information at the species level.

**Phylum**	**Class**	**Order**	**Family**	**Genus**	**Species**	**Species annotation labeled with higher taxa level**
Bacteroidota	Bacteroidia	Bacteroidales	F082	F082	s__uncultured_rumen	s__uncultured rumen Bacteroidales F082 (G)
Bacteroidota	Bacteroidia	Bacteroidales	F082	F082	s__uncultured_bacterium	s__uncultured bacterium Bacteroidales F082 (G)
Bacteroidota	Bacteroidia	Bacteroidales	Marinifilaceae	uncultured	s__uncultured_rumen	s__uncultured rumen Marinifilaceae (F)
Bacteroidota	Bacteroidia	Bacteroidales	Rikenellaceae	Mucinivorans	s__uncultured_bacterium	s__uncultured bacterium Mucinivorans (G)
Bacteroidota	Bacteroidia	Bacteroidales	p-251-o5	p-251-o5	s__uncultured_bacterium	s__uncultured bacterium Bacteroidales p-251-o5 (G)
Bacteroidota	Bacteroidia	Bacteroidales	Marinifilaceae	Odoribacter	s__Odoribacter_sp.	s__Odoribacter sp.
Bacteroidota	Bacteroidia	Bacteroidales	Bacteroidaceae	Bacteroides	s__Bacteroides_fragilis	s__Bacteroides fragilis
Bacteroidota	Bacteroidia	Bacteroidales	Prevotellaceae	Prevotella	s__uncultured_bacterium	s__uncultured bacterium Prevotella (G)
Campilobacterota	Campylobacteria	Campylobacterales	Campylobacteraceae	Campylobacter	s__Campylobacter_hyointestinalis	s__Campylobacter hyointestinalis
Firmicutes	Clostridia	Clostridiales	Clostridiaceae	Sarcina	s__uncultured_bacterium	s__uncultured bacterium Sarcina (G)
Firmicutes	Clostridia	Lachnospirales	Lachnospiraceae	Oribacterium	s__uncultured_bacterium	s__uncultured bacterium Oribacterium (G)
Firmicutes	Negativicutes	Acidaminococcales	Acidaminococcaceae	Phascolarctobacterium	s__Phascolarctobacterium_faecium	s__Phascolarctobacterium faecium
Firmicutes	Clostridia	Oscillospirales	Ruminococcaceae	Ruminococcus	s__Ruminococcus_flavefaciens	s__Ruminococcus flavefaciens
Firmicutes	Negativicutes	Acidaminococcales	Acidaminococcaceae	Succiniclasticum	s__gut_metagenome	s__gut metagenome Succiniclasticum (G)
Firmicutes	Bacilli	Bacillales	Planococcaceae	uncultured	s__uncultured_Firmicutes	s__uncultured Firmicutes Planococcaceae (F)
Firmicutes	Clostridia	Lachnospirales	Lachnospiraceae	Shuttleworthia	s__uncultured_bacterium	s__uncultured bacterium Shuttleworthia (G)
Proteobacteria	Gammaproteobacteria	Enterobacterales	Enterobacteriaceae	Escherichia-Shigella	s__Escherichia_coli	s__Escherichia coli
Proteobacteria	Gammaproteobacteria	Enterobacterales	Enterobacteriaceae	Escherichia-Shigella	s__Shigella_flexneri	s__Shigella flexneri
Proteobacteria	Gammaproteobacteria	Aeromonadales	Succinivibrionaceae	Succinivibrio	s__metagenome	s__metagenome Succinivibrio (G)
Proteobacteria	Gammaproteobacteria	Pseudomonadales	Moraxellaceae	Acinetobacter	s__uncultured_bacterium	s__uncultured bacterium Acinetobacter (G)
Proteobacteria	Gammaproteobacteria	Burkholderiales	Comamonadaceae	Comamonas	s__Comamonas_kerstersii	s__Comamonas kerstersii
Proteobacteria	Gammaproteobacteria	Pseudomonadales	Moraxellaceae	Acinetobacter	s__Acinetobacter_sp.	s__Acinetobacter sp.
Verrucomicrobiota	Verrucomicrobiae	Verrucomicrobiales	Akkermansiaceae	Akkermansia	s__uncultured_organism	s__uncultured organism Akkermansia (G)

Supplementary Table 3. The sequences of full-length 16S rRNA genes (V1-V9) by Oxford nanopore sequencing of the fecal microbiome of 32 captive elephants in Thailand.

Sample	Read number	Mean read length (bp)	Mean read quality	Reads used for clustering
EAA01	60,082	1,449	14	56,581
EAA02	1,851,079	1,439	14	1,729,638
EAA03	97,112	1,437	13.8	91,802
EAA04	68,569	1,433	14	64,731
EAK03	71,108	1,456	14	67,770
EAK05	552,607	1,438	13.9	524,592
EAK06	288,458	1,434	13.9	273,066
EAK09	358,124	1,439	13.9	341,351
EAK10	442,776	1,434	13.9	422,667
EAK11	43,426	1,438	13.9	40,730
EAK12	269,902	1,438	13.9	256,660
EAS15	253,075	1,438	14.1	245,885
EBK01	69,125	1,449	14	67,279
EBK02	95,005	1,452	14	88,276
EBK04	69,452	1,444	14.1	65,074
EBS10	162,816	1,419	13.6	148,775
EBS12	361,945	1,444	13.9	347,886
EBS16	105,444	1,444	14	102,123
EJK07	193,777	1,431	13.9	183,127
EJK08	194,060	1,438	13.8	185,255
EJS01	304,843	1,435	13.9	287,863
EJS02	262,756	1,435	13.9	251,536
EJS03	241,940	1,438	13.9	230,585
EJS04	364,526	1,436	13.9	337,768
EJS05	259,065	1,435	13.9	249,397
EJS06	341,437	1,438	13.9	328,859
EJS07	251,242	1,435	13.8	240,849
EJS08	266,529	1,437	13.9	256,421
EJS09	346,980	1,437	13.9	329,947
EJS11	61,820	1,435	13.7	57,516
EJS13	64,616	1,439	14	57,693
EJS14	71,114	1,439	14	67,062

Supplementary Table 4. Permutational multivariate analysis of variance (PERMANOVA) measures significant differences between microbiome profiles between different sub-categories of elephant-fed diets. Asterisk (*) indicates the significant difference (p-value <= 0.05) between the two sample groups. N/A means not applicable as there is no data for a comparison.

	**Comparison**	**32 samples**		**Baby**		**Juvenile**		**Adult**	
		**R-value**	**P-value**	**R-value**	**P-value**	**R-value**	**P-value**	**R-value**	**P-value**
**Location**	Central-West-Northeast	0.17176	0.016*	N/A	N/A	N/A	N/A	0.49288	0.003*
	Central-West	0.00087	0.411	N/A	N/A	N/A	N/A	0.48942	0.009*
	Central-Northeast	0.09425	0.288	N/A	N/A	N/A	N/A	0.66667	0.189
	West-Northeast	0.22917	0.002*	0.37037	0.106	0.31468	0.149	0.41497	0.239
**Gender**	Male-Female	0.07044	0.193	0.33333	0.199	0.00097	0.413	N/A	N/A
**Feed**	Milk-without	0.63269	0.01*	0.37037	0.106	N/A	N/A	N/A	N/A
	Banana-without	0.22421	0.02*	0.14815	0.416	0.02035	0.333	0.26281	0.167
	Napier grass-without	0.19066	0.008*	-0.04	0.488	0.31468	0.185	0.55926	0.004*
	Native grass-without	0.19066	0.008*	-0.52	1	N/A		0.33640	0.02*
	Pineapple-without	0.13570	0.046*	-0.52	1	0.14118	0.189	0.06363	0.312
	Caryota urens-without	0.16807	0.173	N/A	N/A	0.31468	0.149	0.55926	0.004*
	Sugarcane-without	0.03502	0.329	0.03571	0.42	-0.17241	0.757	N/A	N/A
**Age**	Adult-Baby-Juvenile	0.36490	<0.001*	N/A	N/A	N/A	N/A	N/A	N/A
	Adult-Baby	0.52932	0.002*	N/A	N/A	N/A	N/A	N/A	N/A
	Adult-Juvenile	0.24689	0.001*	N/A	N/A	N/A	N/A	N/A	N/A
	Baby-Juvenile	0.53122	0.006*	N/A	N/A	N/A	N/A	N/A	N/A

Supplement Table 5. Differential metabolic pathways of fecal microbiome of captive Asian elephants in different age classes.

Supplementary Table5A. Significant enriched metabolic pathways of fecal microbiome in baby elephants compared to juveniles.

Class	Subclass	KEGG pathway	Baby: mean rel. freq. (%)	Juvenile: mean rel. freq. (%)	Difference between means	p-values
**1. Metabolism**	1.2 Energy metabolism	ko00910: Nitrogen metabolism	0.6401	0.4899	-0.1502	0.0357
		ko00920: Sulfur metabolism	0.9493	0.7511	-0.1982	0.0359
	1.3 Lipid metabolism	ko00140: Steroid hormone biosynthesis	0.0353	0.0002	-0.0350	0.0364
	1.6 Metabolism of other amino acids	ko00440: Phosphonate and phosphinate metabolism	0.2018	0.1198	-0.0819	0.0366
	1.8 Metabolism of cofactors and vitamins	ko00130: Ubiquinone and other terpenoid-quinone biosynthesis	0.7105	0.3813	-0.3291	0.0494
	1.9 Metabolism of terpenoids and polyketides	ko01053: Biosynthesis of siderophore group nonribosomal peptides	0.1711	0.0114	-0.1597	0.0436
	1.11 Xenobiotics biodegradation and metabolism	ko00621: Dioxin degradation	0.1645	0.0031	-0.1614	0.0425
**3. Environmental Information Processing**	3.2 Signal transduction	ko02020: Two-component system	0.5570	0.3010	-0.2560	0.0435
**4. Cellular Processes**	4.4 Cellular community - prokaryotes	ko05111: Vibrio cholerae pathogenic cycle	0.2072	0.0595	-0.1477	0.0426

Supplementary Table5B. Significant enriched metabolic pathways of fecal microbiome in juvenile compared to baby elephants.

**Class**	**Subclass**	**KEGG pathway**	**Baby: mean rel. freq. (%)**	**Juvenile: mean rel. freq. (%)**	**Difference between means**	**p-values**
**1. Metabolism**	1.1 Carbohydrate metabolism	ko00660: C5-Branched dibasic acid metabolism	2.0407	2.1949	0.1542	0.0389
	1.2 Energy metabolism	ko00710: Carbon fixation in photosynthetic organisms	1.5272	1.7287	0.2015	0.0381
	1.4 Nucleotide metabolism	ko00240: Pyrimidine metabolism	1.1187	1.2288	0.1101	0.0409
		ko00230: Purine metabolism	0.8682	0.8971	0.0289	0.0391
	1.5 Amino acid metabolism	ko00471: D-Glutamine and D-glutamate metabolism	1.9380	2.3214	0.3833	0.0331
		ko00290: Valine, leucine and isoleucine biosynthesis	2.3080	2.6228	0.3148	0.0125
		ko00400: Phenylalanine, tyrosine and tryptophan biosynthesis	1.2615	1.4774	0.2158	0.0373
	1.7 Glycan biosynthesis and metabolism	ko00510: N-Glycan biosynthesis	0.0665	0.1248	0.0583	0.0312
	1.8 Metabolism of cofactors and vitamins	ko00670: One carbon pool by folate	1.6482	1.9149	0.2667	0.0476
		ko00770: Pantothenate and CoA biosynthesis	1.8943	2.1460	0.2517	0.0125
	1.9 Metabolism of terpenoids and polyketides	ko01055: Biosynthesis of vancomycin group antibiotics	2.2871	3.3419	1.0548	0.0439
		ko00900: Terpenoid backbone biosynthesis	1.3060	1.5312	0.2253	0.0347
		ko00908: Zeatin biosynthesis	0.5244	0.6532	0.1288	0.0397
	1.10 Biosynthesis of other secondary metabolites	ko00521: Streptomycin biosynthesis	1.3846	1.8315	0.4469	0.0321
**2. Genetic Information Processing**	2.2 Translation	ko03010: Ribosome	1.4952	1.7938	0.2986	0.0423
		ko00970: Aminoacyl-tRNA biosynthesis	1.5426	1.7831	0.2405	0.0352
	2.3 Folding, sorting and degradation	ko03060: Protein export	1.4623	1.6068	0.1446	0.0357
		ko04141: Protein processing in endoplasmic reticulum	0.0417	0.0637	0.0221	0.0322
	2.4 Replication and repair	ko03430: Mismatch repair	1.5491	1.7437	0.1947	0.0350
		ko03440: Homologous recombination	1.4152	1.6060	0.1909	0.0332
		ko03030: DNA replication	1.1673	1.3376	0.1703	0.0362
		ko03420: Nucleotide excision repair	0.6896	0.8219	0.1323	0.0307
**4. Cellular Processes**	4.2 Cell growth and death	ko04112: Cell cycle - Caulobacter	1.3735	1.6051	0.2315	0.0427
**6. Human Diseases**	6.11 Drug resistance: antimicrobial	ko00312: beta-Lactam resistance	0.0260	0.0831	0.0570	0.0155

Supplementary Table5C. Significant enriched metabolic pathways of fecal microbiome in juvenile compared to adult elephants.

Class	Subclass	KEGG pathway	Juvenile: mean rel. freq. (%)	Adult: mean rel. freq. (%)	Difference between means	p-values
**1. Metabolism**	1.5 Amino acid metabolism	ko00290: Valine, leucine and isoleucine biosynthesis	2.6228	2.4907	-0.1321	0.0229
		ko00300: Lysine biosynthesis	1.6779	1.5712	-0.1067	0.0148
	1.7 Glycan biosynthesis and metabolism	ko00550: Peptidoglycan biosynthesis	1.9767	1.8801	-0.0965	0.0210
	1.8 Metabolism of cofactors and vitamins	ko00730: Thiamine metabolism	1.5370	1.3792	-0.1578	0.0210
	1.9 Metabolism of terpenoids and polyketides	ko00908: Zeatin biosynthesis	0.6532	0.5992	-0.0540	0.0189
		ko00900: Terpenoid backbone biosynthesis	1.5312	1.4839	-0.0473	0.0386
**2. Genetic Information Processing**	2.2 Translation	ko03008: Ribosome biogenesis in eukaryotes	0.0594	0.0570	-0.0024	0.0157
		ko03010: Ribosome	1.7938	1.7015	-0.0923	0.0140
		ko00970: Aminoacyl-tRNA biosynthesis	1.7831	1.6709	-0.1121	0.0315
	2.3 Folding, sorting and degradation	ko03060: Protein export	1.6068	1.5374	-0.0694	0.0199
	2.4 Replication and repair	ko03430: Mismatch repair	1.7437	1.6786	-0.0651	0.0175
		ko03440: Homologous recombination	1.6060	1.5261	-0.0800	0.0210
		ko03030: DNA replication	1.3376	1.2909	-0.0467	0.0327
		ko03420: Nucleotide excision repair	0.8219	0.7909	-0.0311	0.0289
		ko03410: Base excision repair	0.9362	0.9135	-0.0228	0.0339
**5. Organismal Systems**	5.2 Endocrine system	ko04910: Insulin signaling pathway	0.1129	0.0950	-0.0178	0.0277

Supplementary Table5D. Significant enriched metabolic pathways of fecal microbiome in adult compared to juvenile elephants.

Class	Subclass	KEGG pathway	Juvenile: mean rel. freq. (%)	Adult: mean rel. freq. (%)	Difference between means	p-values
**1. Metabolism**	1.1 Carbohydrate metabolism	ko00562: Inositol phosphate metabolism	0.1913	0.2166	0.0253	0.0369
		ko00010: Glycolysis / Gluconeogenesis	1.0939	1.1307	0.0368	0.0378
		ko00630: Glyoxylate and dicarboxylate metabolism	0.7093	0.7643	0.0550	0.0229
		ko00650: Butanoate metabolism	0.7965	0.8679	0.0714	0.0180
	1.11 Xenobiotics biodegradation and metabolism	ko00362: Benzoate degradation	0.2165	0.2605	0.0439	0.0360
	1.2 Energy metabolism	ko00920: Sulfur metabolism	0.7511	0.8201	0.0690	0.0157
	1.3 Lipid metabolism	ko00072: Synthesis and degradation of ketone bodies	1.1675	1.5239	0.3564	0.0246
	1.5 Amino acid metabolism	ko00350: Tyrosine metabolism	0.3177	0.3785	0.0609	0.0252
		ko00310: Lysine degradation	0.2994	0.3793	0.0799	0.0157
		ko00380: Tryptophan metabolism	0.2692	0.3509	0.0817	0.0168
		ko00280: Valine, leucine and isoleucine degradation	0.7044	0.8950	0.1905	0.0220
	1.8 Metabolism of cofactors and vitamins	ko00130: Ubiquinone and other terpenoid-quinone biosynthesis	0.3813	0.4941	0.1128	0.0210
	1.9 Metabolism of terpenoids and polyketides	ko00281: Geraniol degradation	0.2434	0.3920	0.1486	0.0194
**2. Genetic Information Processing**	2.2 Translation	ko03013: RNA transport	0.0513	0.0578	0.0065	0.0180
